# Gender bias in cardiovascular practice guidelines: the example of ischemic heart diseases

**DOI:** 10.1093/eurpub/ckac129.559

**Published:** 2022-10-25

**Authors:** K Bastian, JL Rohmann, M Piccininni, M Kelly Irving, N Bajos

**Affiliations:** 1 IRIS - EHESS - U997 INSERM, Interdisciplinary Institute of Social Issues IRIS, Aubervilliers, France; 2 Institute of Public Health, Charité - Universitätsmedizin Berlin, Berlin, Germany; 3 Center for Stroke Research Berlin, Charité - Universitätsmedizin Berlin, Berlin, Germany; 4 CERPOP-UMR1295, Université de Toulouse III, Toulouse, France

## Abstract

**Background:**

Despite declines in cardiovascular disease mortality, evidence suggests women have a higher mortality and worse prognosis after ischemic events compared with men. This work investigates to what extent clinical recommendations made by cardiovascular guidelines might include sex and gender biases.

**Methods:**

We reviewed the primary literature base underlying the 2019 European Society of Cardiology guidelines’ recommendations on chronic coronary syndromes focusing on sex and gender sensitivity aspects pertaining to ischemic heart diseases. We applied an adapted Cochrane Collaboration appraisal checklist and only included recommendations with direct citation to literature evidence. We used beta regression to model the association between the proportion of female participants within the study population and the year of publication (modeled as cubic polynomial), impact factor, and sex of the first/last author.

**Results:**

In the 20 recommendations that quoted the literature, gender-sensitive propositions were fully absent, while 4 included sex-sensitive statements. Sex- or gender- specific study design or a priori statistical considerations were largely absent in the included publications. The term “gender” was used exclusively to denote biological sex. The included studies published between 1991 and 2019 comprised of more than 2.1 million individuals (29% female). Representation of women as study participants undulated over time. Female sex of first (OR = 1.84, 95%CI= 1.30-2.60) or last (OR = 2.27, 95%CI=1.31-3.92) author were statistically significantly associated with the probability of having more female study participants.

**Conclusions:**

Methodological sex- and gender-sensitive considerations are largely missing in the evidence base used to guide cardiovascular care decision-making in Europe. Women remain underrepresented in the studies from which the guidelines are derived. Studies with female first or last authors were more likely to have better representation of women.

**Key messages:**

• Though differences in morbidity are well-described, current European cardiovascular recommendations lack sex and gender sensitivity, and women remain underrepresented in the underlying evidence base.

• To what extent the clinical cardiovascular recommendations may or may not be valid for groups not well-represented in the underlying evidence base remains unknown.

